# Predictive ability of serum osmolarity for contrast-induced nephropathy after elective percutaneous coronary intervention: Are we having a new target?

**DOI:** 10.1186/s43044-025-00620-8

**Published:** 2025-02-21

**Authors:** Ahmad Samir, Aly Radwan, Hossam Elhossary, Yasser Baghdady

**Affiliations:** Kasr AlAiny Faculty of Medicine, Cairo University, Cairo, Egypt

**Keywords:** Contrast-induced nephropathy CIN, Osmolarity, Dehydration, Diabetes mellitus, Loop diuretics

## Abstract

**Background:**

Contrast-induced nephropathy (CIN) remains a serious complication following percutaneous coronary intervention (PCI), often leading to poor outcomes. Although the overall incidence of CIN is low, the risk can be significantly higher in certain susceptible cohorts.

**Results:**

This prospective observational analytic study enrolled 174 consecutive eligible patients. The study selectively included diabetic patients with heart failure who are receiving regular diuretic therapy, being scheduled for elective coronary angiography (CAG) and/or PCI. CIN occurred in 24.7% of the study participants. CIN patients had significantly higher baseline osmolarity compared to those who did not develop CIN. After adjusting for other factors, pre-procedure osmolarity ≥ 302.3 mOsm/L, higher CHA_2_DS_2_VA score, and larger contrast volume proved to be independent predictors for CIN with an odds ratio and 95% confidence interval of 7.07 (2.47–20.26), 3.99 (2.02–7.9), and 1.01 (1.0–1.014), respectively.

**Conclusions:**

In patients at high risk for CIN, serum osmolarity can serve as a practical stratification tool for CIN risk before elective CAG or PCI. Future studies should evaluate whether targeting a specific pre-procedural osmolarity threshold can reduce the risk of post-PCI CIN.

**Graphical abstract:**

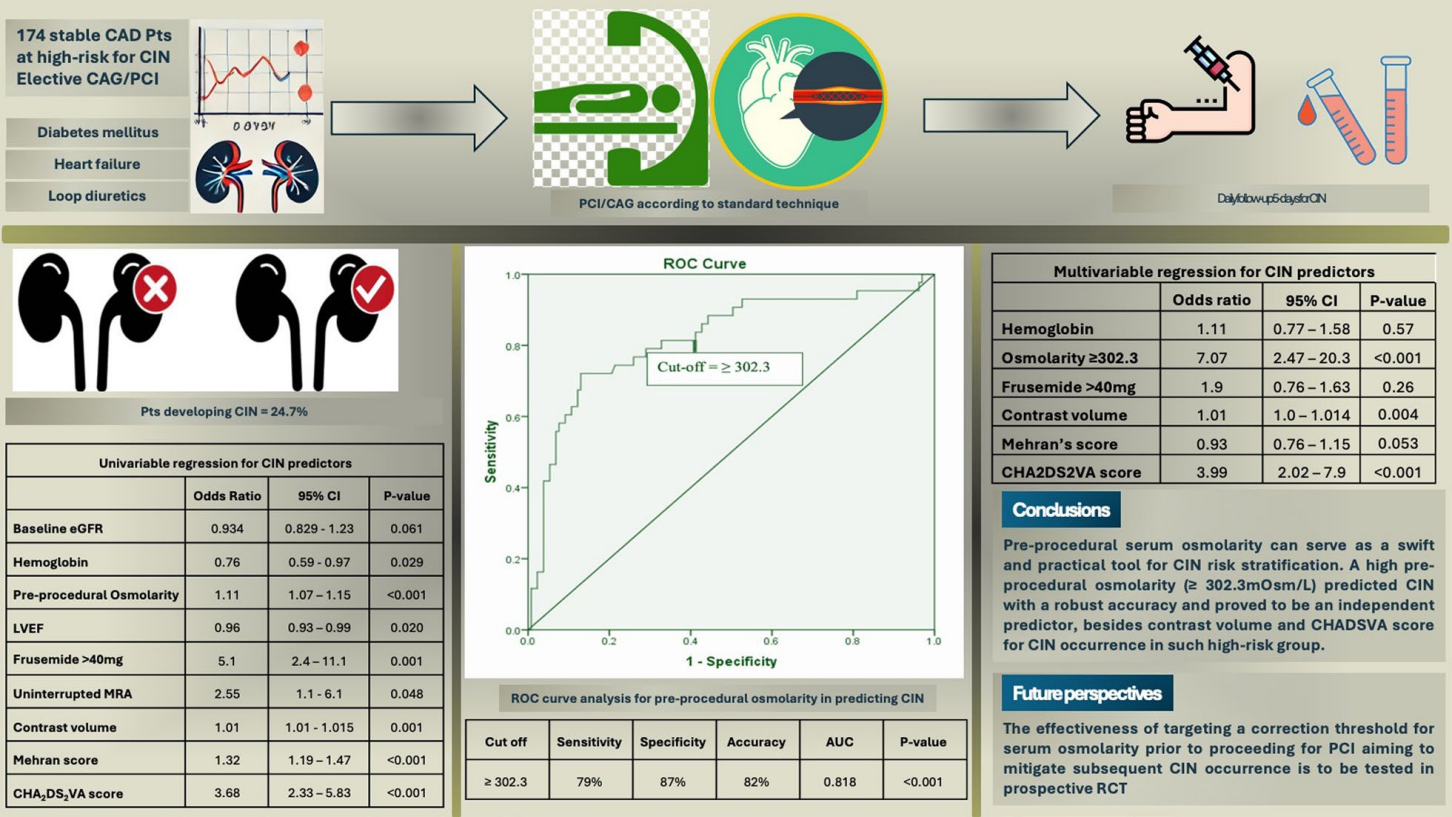

## Background

Contrast-induced nephropathy (CIN) or often termed contrast-associated acute kidney injury (CA-AKI) is defined as new renal impairment following exposure to iodinated contrast media; and can occasionally lead to serious complications including renal failure or death [1–4]. While the overall incidence of contrast-induced nephropathy (CIN) in the general population is relatively low, ranging from 1 to 2%, certain susceptible populations may have a significantly higher risk, with rates reaching up to 25% [2, 5–8].

Given the serious consequences of CIN, several preventive interventions were evaluated but proved ineffective, such as administrations of N-acetyl cysteine or intravenous sodium bicarbonate [1, 9]. On the other hand, adequate peri-procedural hydration remains one of the most useful and recommended preventive interventions [8, 10], highlighting the critical impact of hydration status on CIN risk. In several large studies, compared to euhydrated patients, those with dehydration have been found to have up to fivefold higher risk of developing CIN [9, 11]. Therefore, identifying a measurable surrogate for the hydration status may provide a useful tool for pre-procedural risk stratification, and probably may identify a therapeutic target for correction for susceptible cohorts deemed at high risk.

## Methods

This was a prospective observational analytic study conducted in a tertiary cardiac center. The study included coronary artery disease (CAD) patients scheduled for elective coronary angiography (CAG) or percutaneous coronary intervention (PCI), from both genders. To selectively include patients at high risk for CIN, inclusion criteria comprised confirmed diagnosis of DM, established diagnosis of HF, and being on regular loop diuretics for at least 15 days before recruitment. The diagnosis of DM was established by either a hemoglobin A1c (HbA1c) level of > 6.5%, a fasting plasma glucose level of > 126 mg/dL, repeated random blood glucose (RBG) level of > 200 mg/dL (11.1 mmol/L) in the presence of the classic signs and symptoms of hyperglycemia, or prior diagnosis with DM with ongoing antidiabetic medications [12]. The diagnosis of HF was based on the universal definition and classification of heart failure, including HF-eligible phenotypes with reduced, mildly reduced, or preserved ejection fraction [13]. Regular loop diuretics therapy was defined as at least once daily dosing of frusemide, torsemide, or bumetanide for the prior 15 days. For the purpose of standardization and unification of dose expression in the statistical analysis, oral bumetanide 1mg and torsemide 10 mg were equated to oral frusemide 40 mg [14]. The exclusion criteria comprised history of acute myocardial infarction (AMI) within 30 days, chronic kidney disease (CKD) with estimated glomerular filtration rate (eGFR) ≤ 60 ml/min/1.7m^2^ or those on regular hemodialysis, decompensated HF or cardiogenic shock (NYHA class IV or Killip class IV), pregnancy, or breastfeeding.

The study protocol, methodology as well as the patients’ informed consent forms were reviewed by the institutional research scientific and ethics committee, ensuring their accordance with the Declaration of Helsinki. All study documentations were approved by the Research Ethical Committee, Faculty of Medicine, Cairo University, and registered as [MS-4522021]. After ensuring eligibility, all recruited patients provided written informed consent for study participation and subsequent publication of anonymized results. The statistical analysis was performed on anonymized data after concealing any identifier information to ensure patients’ confidentiality.

The sample size was calculated using MedCalc Statistical Software version 14.10.2 (MedCalc Software bvba, Ostend, Belgium; http://www.medcalc.org; 2014). The calculation utilized a type I error of 0.05 and a type II error of 10%, targeting an area under the curve (AUC) of 0.7 while presuming a CIN prevalence of at least 15% in the selected high-risk patient profile.

### Data collection

All eligible patients underwent baseline clinical evaluation, including age, gender, body weight, height, prior history of CAD, and/or coronary revascularization as well as the conventional risk factors for CAD. HF assessment was represented in Killip classification [15], where Killip class I indicated no current signs of heart failure, class II represented patients with S3, basal crepitations, or signs suggesting elevated venous pressure, class III represented patients with pulmonary edema, while class IV indicated patients with cardiogenic shock (which was an exclusion criterion). After being subjected to general and cardiac examination, patients were interrogated for the pre-procedure regular medical therapies including antiplatelets, anticoagulants, beta-adrenergic blockers (BB), statins, angiotensin-converting enzyme inhibitors (ACEi), angiotensin receptor blockers (ARBs), angiotensin receptor-neprilysin inhibitor (ARNI), mineralocorticoid receptor antagonist (MRA), and the antidiabetic treatments including insulin and oral hypoglycemics (with special emphasis on metformin and sodium-glucose cotransporter 2 inhibitors (SGLT2i)). Special attention was given to the loop diuretics including type and dose; then, their expression was unified into frusemide dose equivalents (as previously mentioned). Left ventricular (LV) ejection fraction (EF), end-diastolic diameter (EDD), and end-systolic diameter (ESD) were revised and registered from a recent (within 2 months) echocardiographic examination.

Pre-procedural laboratory assessment was routinely performed ensuring the evaluation of: (1) Serum sodium (Na), serum potassium (K), and RBG tested within 120 min from the procedure; (2) serum urea, serum creatinine (SCr), and eGFR (calculated according to Cockroft–Gault formula) [16]; and (3) serum osmolarity (mOsm/L) was calculated as 2(Na) + Glucose/18 + Urea/5.6 [17].

The CAG and/or PCI were performed according to the standard technique utilizing non-ionic iso-osmolar contrast in all patients. Pre-procedural hydration was according to the operator's discretion yet ensuring that serum osmolarity was equated immediately before the PCI (after hydration) in these cases. Procedural data including procedure type (CAG only or PCI), procedural time, and contrast media volume (CMV) were reviewed and reported. Post-procedure, Mehran’s risk score [18], and the CHA_2_DS_2_VA score (congestive heart failure or systolic dysfunction, hypertension, age ≥ 75y, diabetes mellitus, stroke or systemic embolization, vascular disease, and age 65-to-74y) [5] were calculated and registered for all patients. Notably, the point for sex category was purposefully dropped from the CHA_2_DS_2_VASc acknowledging that the eGFR calculation already counted for the female sex.

SCr and estimated eGFR were systematically followed up once daily or more frequently as appropriate till the 5th day post-procedure (if CIN was not met), or till the time of recovery or hemodialysis (in cases of CIN). Patients showing any trend toward rising SCr, even if below the diagnostic threshold, were followed for a longer period. The CIN diagnosis was established when SCr rose by ≥ 25% from baseline, according to the kidney disease improving global outcomes (KDIGO) definition for CA-AKI [16]. Thereby, according to the post-procedural follow-up of SCr, patients were categorized into those who did develop CIN and those who did not. Subsequently, clinical correlates and predictors for CIN were sought.

### Statistical analysis

Data were collected and tabulated, then analyzed utilizing Statistical Package for Social Sciences (SPSS 26.0 for Windows, SPSS Inc., Chicago, Illinois). Continuous data were assessed for normality using the Kolmogorov–Smirnov test, where normally distributed data were presented as mean ± standard deviation (SD), while non-normally distributed data were presented as median and interquartile range (IQR). Categorical data were summarized as frequencies and percentages and compared between groups using Chi-square or Fisher's exact test. On the other hand, continuous variables were compared using Student's t-test or Mann–Whitney U test as appropriate. Pearson's correlation was used for normally distributed variables, while Spearman's correlation was used for non-normal variables. Receiver operating characteristic (ROC) curve analysis was used to evaluate the optimal cutoff value, sensitivity, and specificity for the serum osmolarity in predicting CIN. Univariable logistic regression was conducted; then, significant predictors were pooled into a multivariable logistic regression [ENTER METHOD] to identify independent predictors for CIN in this study group. A two-sided p value less than 0.05 was considered statistically significant.

## Results

This study prospectively recruited consecutive 174 eligible stable CAD patients scheduled for CAG and/or PCI from August 2022 to January 2024. Eligibility criteria were tailored to recruit patients whose clinical characteristics put them at higher risk for CIN. The mean age of the study group was 58.6 ± 9.8 years, with a male predominance (113 patients, 65%). CIN occurred in 43 patients (24.7%) of the recruited cohort. Baseline characteristics of the entire cohort and comparisons between patients who developed CIN and those who did not are detailed in Table 1. The baseline demographic data between patients who did and those who did not develop CIN were comparable, except for a higher incidence of hypertension (HTN), chronic obstructive pulmonary disease (COPD), HF Killip class ≥ 2, and HF with reduced ejection fraction (HFrEF) among the CIN group.

**Table 1:** Baseline characteristics for the study group

	All patients (*n* = 174)	CIN patients (*n* = 43)	CIN-free patients (*n* = 131)	*p* value^*^
Age (years)	58.61 ± 9.8	63.47 ± 11.63	57.02 ± 8.58	< 0.001
Male gender	113 (65%)	25 (58%)	88 (67%)	0.281
Weight (kg)	88.35 ± 9.48	89.51 ± 8.65	87.97 ± 9.74	0.356
Height (cm)	170.91 ± 7.24	167.74 ± 5.84	171.95 ± 7.37	0.001
Body mass index (kg/m^2^)	30.36 ± 3.84	31.91 ± 3.74	29.86 ± 3.75	0.002
Body surface area (m^2^)	2.05 ± 0.122	2.04 ± 0.107	2.05 ± 0.127	0.660
Hypertension	106 (61%)	34 (79%)	72 (55%)	0.005
Smoking				
Non-smoker	46 (26%)	10 (23%)	36 (27%)	
Ex-smoker	92 (53%)	24 (56%)	68 (52%)	0.855
Active smoker	36 (21%)	9 (21%)	27 (21%)	
HF Killip class ≥ 2	58 (33%)	23 (54%)	35 (27%)	0.001
HFpEF (≥ 50%)	23 (13%)	8 (19%)	15 (12%)	0.012
HFmEF (41–49%)	20 (12%)	9 (21%)	11 (8%)	
HFrEF (≤ 40%)	15 (9%)	6 (14%)	9 (7%)	
COPD	43 (25%)	18 (42%)	25 (19%)	0.003
Prior CCS	100 (58%)	21 (49%)	79 (60%)	0.334
Prior ACS	63 (36%)	20 (47%)	43 (33%)	0.195
Prior PCI	1 (1%)	1 (2%)	0	0.214
Prior CABG	10 (6%)	0	10 (8%)	0.161
LVEDD (cm)	5.31 ± 0.492	5.39 ± 0.624	5.28 ± 0.439	0.202
LVESD (cm)	3.54 ± 0.644	3.71 ± 0.777	3.49 ± 0.586	0.046
LVEF (%)	57.67 ± 10.21	54.47 ± 11.24	58.73 ± 9.66	0.017
ePASP (mmHg)	28.22 ± 6.1	29.7 ± 6.28	27.73 ± 5.98	0.066
Aspirin	144 (83%)	36 (84%)	108 (82%)	0.847
Clopidogrel	86 (49%)	24 (56%)	62 (47%)	0.334
Ticagrelor	25 (14%)	6 (14%)	19 (14%)	0.929
Atorvastatin	70 (40%)	21 (49%)	49 (37%)	0.185
Rosuvastatin	69 (40%)	13 (30%)	56 (43%)	0.146
High-intensity statins	78 (44%)	17 (39.5%)	61 (46.6%)	0.541
ACEI/ARB/ARNI	102 (59%)	29 (67%)	73 (56%)	0.176
Frusemide^$^				
≤ 40 mg	135 (77.6%)	23 (53.5%)	112 (85.5%)	< 0.001
> 40 mg	39 (22.4%)	20 (46.5%)	19 (14.5%)	
MRA	27 (15%)	11 (26%)	16 (12%)	0.048
Insulin	119 (68%)	28 (65%)	91 (70%)	0.595
OHG therapies	71 (41%)	21 (449%)	50 (38%)	0.289
SGLT2i	51 (29%)	15 (35%)	36 (28%)	0.355
Metformin	37 (21.3%)	10 (23.3%)	27 (20.6%)	0.71
Hemoglobin (g/dl)	12.82 ± 1.41	12.42 ± 1.49	12.95 ± 1.37	0.030
Serum creatinine (mg/dl)	1.06 ± 0.275	1.08 ± 0.414	1.06 ± 0.212	0.641
Urea (mg/dl)	35.34 ± 9.84	39.21 ± 10.01	34.08 ± 9.49	0.003
Sodium (mmol/L)	138.96 ± 5.46	142.67 ± 4.94	137.74 ± 5.07	< 0.001
Potassium (mmol/L)	3.92 ± 0.444	3.98 ± 0.486	3.9 ± 0.429	0.280
Glucose (mg/dl)	171.6 ± 69.1	212.77 ± 64.32	158.11 ± 65.36	< 0.001
Osmolarity (mOsm/L)	293.77 ± 13.1	304.17 ± 11.77	290.35 ± 11.66	< 0.001

*Denoting statistical significance between CIN- and CIN-free patients

^$^Equating torsemide 10mg or bumetanide 1mg to frusemide 40mg. Categorical variables are represented as frequency (%), while continuous variables are represented as mean ± SD or median (IQR) as appropriate

ACEI: Angiotensin-converting enzyme inhibitors; ACS: Acute coronary syndrome; ARB: Angiotensin receptor blockers; ARNI: Angiotensin receptor-neprilysin inhibitor; CABG: Coronary artery bypass graft; COPD: Chronic obstructive pulmonary disease; eGFR: Estimated Glomerular filtration rate; ePASP: Estimated pulmonary artery systolic pressure; HF: Heart failure; HFrEF: Heart failure with reduced ejection fraction; HFpEF: Heart failure with preserved ejection fraction; HFmrEF: Heart failure with mildly reduced ejection fraction; CCS: Chronic coronary syndrome; LVEDD: Left ventricular end-diastolic diameter; LVESD: Left ventricular end-systolic diameter; LVEF: Left ventricular ejection fraction; PCI: Percutaneous coronary intervention; MRA: Mineralocorticoid receptor antagonist; OHG: Oral hypoglycemic; SGLT2i: Sodium-glucose cotransporter 2 inhibitors

Notably, patients who developed CIN were more likely to be on uninterrupted MRA therapy and to be on high doses of loop diuretics (defined as > 40 mg of furosemide (or equivalent) daily). The odds ratios OR (95% confidence interval (CI)) for patients on MRA and on > 40 mg of furosemide to have CIN were 2.55 (1.1–6.1) and 5.1 (2.4–11.1), respectively. Contrarily, being on SGLT2i or metformin showed no significant impact on CIN occurrence.

In the pre-procedural laboratory assessment, serum urea, sodium, RBG, and osmolarity were significantly higher while hemoglobin was significantly lower among the patients who developed CIN compared to those who did not.

In the analysis of the procedural and post-procedural data, the majority of CIN patients had longer procedural times and significantly larger CMV. Also, compared to those who did not develop CIN, CIN patients had significantly higher median Mehran’s score (8 (3–17) vs. 4 (3–18)) and CHA_2_DS_2_VA score (4 (2–6) vs. 3 (1–6)), with *p *< 0.001 for both. The rest of the procedural data are detailed in (Table 2).

**Table 2:** Procedural and post-procedural data of the study group

	All patients (*n* = 174)	CIN patients (*n* = 43)	CIN-free patients (*n* = 131)	*p* value^*^
Contrast volume (ml)	150 (50–500)	220 (50–500)	100 (50–500)	< 0.001
Procedural time (min)	30 (10–90)	40 (10–90)	25 (10–90)	< 0.001
Mehran’s score	5 (3–18)	8 (3–17)	4 (3–18)	< 0.001
CHA_2_DS_2_VA score	3 (1–6)	4 (2–6)	3 (1–6)	< 0.001
Post-procedural FU data				
Highest SCr	1.1 (0.56–6.8)	1.4 (0.7–6.8)	1.02 (0.56–1.56)	< 0.001
Lowest eGFR	82.4 (12.4–120)	62.7 (12.4–116.2)	91.3 (43.2–120)	< 0.001
Increment (%) in SCr	3.8 (− 40–106.1)	30 (25–106.1)	0 (− 40–18.2)	< 0.001

*Denoting statistical significance between CIN- and CIN-free patients

Categorical variables are represented as frequency (%), while continuous variables are represented as mean ± SD or median (IQR) as appropriate

CHA_2_DS_2_VA: Congestive heart failure or systolic dysfunction, hypertension, age ≥ 75y, diabetes mellitus, stroke or systemic embolization, vascular disease, and age 65-to-74y; eGFR: Estimated glomerular filtration rate; FU: Follow-up, SCr: Serum creatinine

**Fig. 1:**
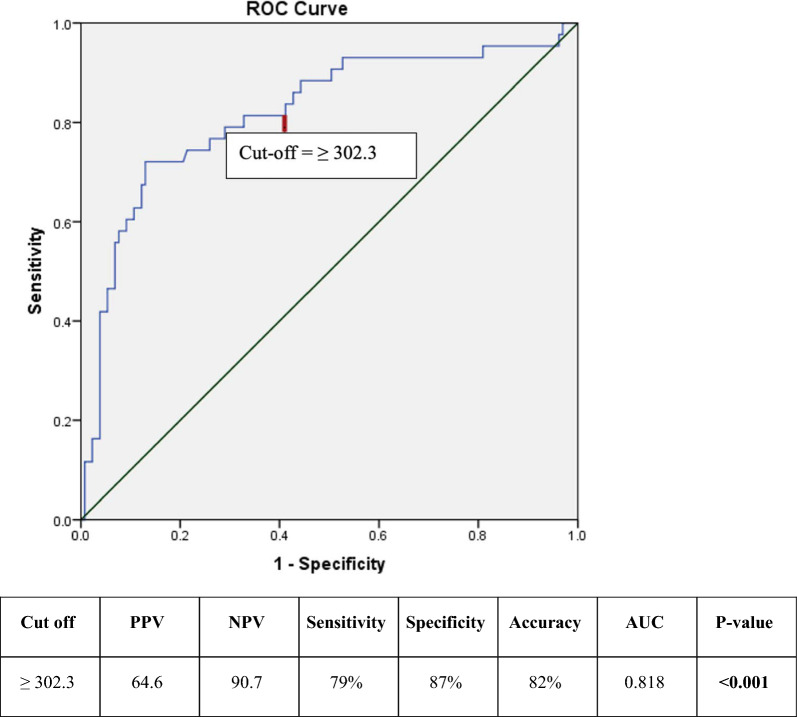
ROC curve for osmolarity predictive ability for CIN among diabetic patients on regular loop diuretics

A ROC curve was constructed to evaluate the ability of the pre-procedural serum osmolarity to predict CIN (Fig. 1). At a cutoff value of 302.3 mOsm/kg, serum osmolarity predicted CIN with a sensitivity of 79.1%, specificity of 87.2%, and AUC of 0.818 (95% CI for AUC: 0.738–0.899). Binary logistic regression analysis was performed to evaluate significant predictors for CIN occurrence (Table 3). In the univariate regression analysis, hemoglobin, serum osmolarity, LVEF, daily frusemide  > 40 mg/day, MRA, CMV, Mehran’s score, and CHA2DS2VA score were significant predictors for CIN. In the multivariate regression analysis, serum osmolarity ≥ 302.3, CMV, and CHA2DS2VA score were identified as significant independent predictors after adjustment for other predictors.

**Table 3:** Univariable and multivariable regression analysis to detect the possible predictors of CIN

	OR	95% CI	*p* value
	Univariable regression		
Baseline SCr	1.5	0.47–4.79	0.49
Hemoglobin	0.76	0.59–0.97	0.029
Osmolarity	1.11	1.07–1.15	< 0.001
eGFR	0.934	0.829–1.23	0.061
Age	1.105	0.987–1.151	0.092
LVEF	0.96	0.93–0.99	0.020
Frusemide > 40mg	5.1	2.4–11.1	0.001
MRA	2.55	1.1–6.1	0.048
Contrast volume	1.01	1.01–1.015	0.001
Mehran score	1.32	1.19–1.47	< 0.001
CHA_2_DS_2_VA score	3.68	2.33–5.83	< 0.001
	Multivariable regression		
Hemoglobin	1.11	0.774–1.58	0.57
Osmolarity 302.3	7.07	2.47–20.26	< 0.001
Frusemide > 40mg	1.9	0.76–1.63	0.26
Contrast volume	1.01	1.0–1.014	0.004
Mehran’s score	0.93	0.76–1.15	0.053
CHA_2_DS_2_VA score	3.99	2.02–7.9	< 0.001

CHA_2_DS_2_VA: Congestive heart failure or systolic dysfunction, hypertension, age ≥ 75y, diabetes mellitus, stroke or systemic embolization, vascular disease, and age 65-to-74y; CI: Confidence interval; eGFR: Estimated glomerular filtration rate; LVEF: Left ventricular ejection fraction; MRA: Mineralocorticoid receptor antagonist; OR: Odd ratio; SCr: Serum creatinine

## Discussion

CIN is defined as a new impairment in renal function following intravascular administration of iodinated contrast media, typically indicated by a rise in SCr of > 0.5 mg/dL or by ≥ 25% from baseline values [1, 3, 10]. Despite the improvements in iodinated contrast agents, the incidence of CIN can be as high as 25% in specific high-risk groups [5, 6]. Furthermore, CIN can lead to serious complications, including renal failure and death [2], underscoring the importance of risk stratification to provide timely and appropriate preventive interventions to those at higher risk [6, 7].

Evidence is established that pre-existing CKD, DM, HF, hypovolemia, hypotension, and advanced age can substantially elevate the risk of CIN [2, 7, 8]. Unfortunately, several of these conditions may occur alongside advanced CAD, either due to common pathophysiological mechanisms or for a potential cause-resultant relationship [19]. Hence, patients scheduled for CAG and/or PCI who have several of these factors become inherently at elevated risk for CIN. Additionally, the traditional practice of fasting for several hours before cardiac catheterization (promoting volume depletion) in such susceptible patients can exacerbate CIN risk even more [2, 6].

Among various measures evaluated for CIN prevention, peri-procedural hydration has proved to be among the most effective strategies, highlighting the detrimental impact of hydration/dehydration on CIN risk [9, 20]. Compared to euhydrated patients, those with hyperosmolar dehydration were consistently found to have a fivefold higher risk of developing CIN [20, 21]. Hydration–dehydration status is typically represented by measuring serum osmolality (in mOsm/Kg). As an alternative to the directly measured osmolality, serum osmolarity (mOsm/L) is a validated parameter that can be easily calculated from serum chemistry and electrolytes, making it a prevalent tool in clinical practice [17]. Classically, serum osmolarity is maintained within a narrow range of normality (275–295 mOsm/L) through rapid physiological adjustments of total body water to compensate for the changes in serum solutes. However, external factors such as prolonged fasting, diuretics, glycosuria, HF, and HF medications may disturb serum osmolarity, which “at least hypothetically” may enhance the risk of CIN.

In this prospective observational analytic study, 174 eligible patients undergoing elective CAG/PCI in a tertiary cardiac center were recruited. The eligibility criteria were tailored to recruit high-risk patients for CIN, hence selectively recruited patients with DM and HF who had been on regular loop diuretics for at least 15 days before recruitment. Although CKD patients are also at elevated risk for CIN, they were purposefully excluded from recruitment for the current study because ideally, they should receive overnight intravenous hydration, which would significantly impact the pre-procedure serum osmolarity.

The mean age of the study group was 58.6 ± 9.8 years, with a male predominance. Among this high-risk cohort, CIN occurred in 43 patients (24.7%). Compared to the CIN-free group, patients who developed CIN were significantly older and had a higher likelihood of HTN, symptomatic HF, and COPD. Also, CIN patients had significantly lower hemoglobin and LVEF levels, while they had significantly higher RBG, serum urea, and serum sodium levels, compared to the CIN-free patients. Additionally, CIN patients underwent longer procedures and received larger CMV.

Most notably, CIN patients were characterized by a significantly higher baseline serum osmolarity compared to the CIN-free group (304.17 ± 11.77 vs. 290.35 ± 11.66, *p *< 0.001). In the ROC curve analysis, a cutoff serum osmolarity value of ≥ 302.3 mOsm/L predicted CIN with an AUC of 0.818, a sensitivity of 79.1%, and a specificity of 87.2%.

In the univariate logistic regression analysis, lower hemoglobin levels, lower LVEF, higher Mehran’s score, larger CMV, higher CHA_2_DS_2_VA score, frusemide ≥40mg, MRA therapy, as well as higher serum osmolarity were identified as significant predictors for CIN occurrence. However, after adjusting for confounding variables in the multivariable regression analysis, only serum osmolarity > 302.3 mOsm/L (OR (95% CI): 7.07 (2.47–20.26)), contrast volume (1.01 (1.0–1.014)), and higher CHA_2_DS_2_VA score (3.99 (2.02–7.9)) remained statistically significant as independent predictors for CIN.

Generally, the use of iso-osmolar or low-osmolar contrast (in preference to hyperosmolar agents) and conscious minimization of the CMV have been proved to mitigate CIN risk in patients undergoing CAG/PCI [6]. Nevertheless, serum osmolarity seems to have a critical impact on CIN risk, where patients with abnormally high serum osmolarity, such as those with DM or chronic HF receiving diuretics, are at substantially higher risk for CIN. Although the underlying mechanism may not be fully elucidated, the iodinated contrast media often increase the already high osmotic load on the kidneys, reduce the renal blood flow and the GFR, and allow for the accumulation of contrast molecules within the kidney, exacerbating kidney injury [4]. Other postulated mechanisms for CIN include activation of endothelin and other vasoconstrictor mediators, enhancing the generation of reactive oxygen species leading to lipid peroxidation and tubular injury, impairment of tubular endothelium mitochondrial function, and stimulation of cytokines and local inflammatory reactions [3, 4].

In another study conducted by Kanbay et al. [21], patients who developed CIN had higher baseline serum osmolarity. Their findings demonstrated that the excess osmolarity in the CIN group was primarily attributed to the increased sodium (by 25.2%) and blood urea nitrogen (by 38.2%) compared to the CIN-free group [21]. Similarly, Yildiz et al. evaluated 768 STEMI patients undergoing PCI and demonstrated that pre-procedural serum osmolarity was a significant independent predictor for CIN. They also reported that other independent predictors for CIN included CMV, hemoglobin levels, LVEF, and baseline SYNTAX II score [22]. Arguably, the strong correlation observed between the SYNTAX II score and CIN risk supports the theory of pan-vascular disease, whereas the more advanced CAD (indicated by a higher SYNTAX score) is paralleled by a decline in the renal reserve to tolerate the iodinated contrast, mirroring an advanced renovascular pathology [23, 24].

On the other hand, Mehran’s score is one of the most validated and trusted tools for predicting CIN risk in clinical practice [18]. In this study, a higher Mehran score was a significant predictor for CIN in univariate regression analysis with an OR of 1.32. However, after adjustment for other relevant variables in multivariate regression analysis, Mehran’s score was not a significant independent predictor for CIN. A plausible explanation for this finding is the selective recruitment of a very specific patient cohort (DM and HF on regular diuretics), which might have led to a distinct pattern of CIN occurrence compared to the general non-selected cohorts.

Although the CHA_2_DS_2_VASc score was originally developed to predict stroke risk in atrial fibrillation (AF) patients, its utility has expanded to include several other cardiovascular adverse outcomes. This broader applicability stems from the score's components, which comprehensively reflect the disease markers for atherosclerotic cardiac and vascular disease. Several recent studies have demonstrated the CHA_2_DS_2_VASc score's ability to predict major adverse events in CAD patients with and without AF, including myocardial infarction, heart failure hospitalization, and death [25–28]. A higher CHA_2_DS_2_VASc score has consistently been correlated with an elevated risk of these events, highlighting its potential role for stratification beyond stroke. Interestingly, the CHA_2_DS_2_VASc score has also shown an emerging ability for predicting CIN after PCI [5, 29, 30]. In the selected high-risk group recruited in this study, the gender-neutral CHA_2_DS_2_VA score was a significant independent predictor for CIN with an OR of 3.99.

To wrap up, in cohorts at an elevated baseline CIN risk, such as patients with DM, HF, and on regular loop diuretics, pre-procedure serum osmolarity can be a helpful tool for stratification of the post-PCI CIN risk. When serum osmolarity exceeds 302.3 mOsm/L, the risk of developing CIN increases exponentially. It remains intriguing whether targeting a specific pre-procedural serum osmolarity level can effectively reduce the risk of CIN. A future prospective randomized study is recommended to evaluate whether programmed IV hydration targeting a specific serum osmolarity level (for example, < 300 mOsm/L) can effectively and safely reduce CIN risk in high-risk patients compared to the discretionary current practice of empirical IV hydration. Such a design can be broader in eligibility and include those with and without pre-existing CKD.

Study limitations.

Among the limitations of this study is that while SCr was regularly monitored for 5 days (covering the peak incidence of CIN), cases with late-developing CIN might have been missed. However, to minimize underdiagnosis, patients showing any rise in SCr (even if below the diagnostic threshold) were followed for a longer period. Another limitation was the use of the relative increase in SCr of ≥ 25% from baseline as the threshold for CIN, rather than an absolute rise of > 0.5 mg/dL, where occasionally the two definitions disagree (~ 10% of cases). Nevertheless, it is well-established that patients with diabetes mellitus (DM), heart failure (HF), and those on regular loop diuretics frequently have some degree of renal impairment (manifest or subtle). In such cohorts, the definition considering baseline SCr (relying on a 25% increase from baseline) was found to be more correlated with clinical outcomes than using a fixed absolute rise of 0.5 mg/dL [18]. Although CKD patients are also at higher risk for CIN, they were excluded from this study because they ideally should receive overnight IV hydration before CAG/PCI procedures, which would impact osmolarity assessment and the study's concept. Therefore, the investigators avoided recruiting patients with eGFR < 60 mL/min/1.73 m^2^, believing it would have been unethical to deprive this high-risk population of hydration. However, the future clinical perspective derived from this proof-of-concept study could be applied to CKD patients to determine whether targeting a specific osmolarity threshold through pre-procedure hydration can effectively reduce CIN risk.

## Conclusions

The risk of CIN varies depending on patient characteristics, with certain subsets at significantly higher risk. Pre-procedural serum osmolarity was an independent predictor of CIN. Systematic assessment of pre-procedural serum osmolarity can serve as a swift and practical tool for stratifying CIN risk. Future studies should evaluate the role of an osmolarity therapeutic target in effectively reducing CIN incidence.

## Data Availability

No datasets were generated or analyzed during the current study.

## Abbreviations

ACEi: Angiotensin-converting enzyme inhibitors
AF: Atrial fibrillation
AMI: Acute myocardial infarction
ARB: Angiotensin receptor blockers
ARNI: Angiotensin receptor-neprilysin inhibitor
AUC: Area under the curve
AUC: Area under the curve
BB: Beta-adrenergic blockers
BUN: Blood urea nitrogen
CA-AKI: Contrast-associated acute kidney injury
CAD: Coronary artery disease
CAG: Coronary angiography
CBC: Complete blood count
CHA_2_DS_2_VA: Congestive heart failure or systolic dysfunction, hypertension, age ≥ 75y, diabetes mellitus, stroke or systemic embolization, vascular disease, and age 65-to-74y
CI: Confidence interval
CIN: Contrast-induced nephropathy
CKD: Chronic kidney disease
CMV: Contrast media volume
COPD: Chronic obstructive pulmonary disease
DM: Diabetes mellitus
EDD: End-diastolic diameter
EF: Ejection fraction
eGFR: Estimated glomerular filtration rate
ESD: End-systolic diameter
ESRD: End-stage renal disease
HbA1c: Hemoglobin A1c (glycated hemoglobin)
HF: Heart failure
HFmrEF: Heart failure with mildly reduced ejection fraction
HFpEF: Heart failure with preserved ejection fraction
HFrEF: Heart failure with reduced ejection fraction
HTN: Hypertension
KDIGO: Kidney disease improving global outcomes
LV: Left ventricular
mOsm/L: Milli-osmoles per liter
MRA: Mineralocorticoid receptor antagonist
OR: Odds ratios
PCI: Percutaneous coronary intervention
RBG: Random blood glucose
SCr: Serum creatinine
SGLT2i: Sodium-glucose cotransporter 2 inhibitors
